# Trends in fertility preference implementation among selected Eastern African countries

**DOI:** 10.12688/f1000research.22064.1

**Published:** 2020-02-03

**Authors:** Vincent Otieno, Alfred Agwanda Otieno, Anne Khasakhala

**Affiliations:** 1 Population Studies and Research Institute, University of Nairobi, Nairobi, Nairobi, 00100, Kenya

**Keywords:** Fertility Preference, Degree of Preference Implementation Index, Wanted Fertility, Eastern Africa

## Abstract

**Background: **There has been continuous debate among scholars regarding fertility transition in Africa. Two conclusions emerge: slow pace of decline because of weak facilitating social programs and high demand for large families amidst weak family planning programs. Accelerated fertility decline is expected to occur if there is both substantial decline in desired fertility and increased level of preference implementation. Despite these conclusions, there are also emergent exceptions in Africa, even among the Eastern African countries. Our motivation for the study of this region therefore lies in this context. First, the East African countries share some similarities in policy framework. Secondly, Rwanda and Kenya appear as exceptional in the drive towards accelerating further fertility decline. Fertility change therefore in any one country may have implications in the neighbouring country due to the commonalities especially in language, cultural traits, diffusion and spread new models of behaviour.

**Methods:** With the utilization of DHS data, we analyse trends overtime in two specific features that scholars have indicated to slow or increase fertility decline. Using Bongaarts supply-demand framework, we first deduce trends in fertility preferences among women of reproductive age (15-49 years) and second, the extent to which women have been able to implement their fertility preferences during the course of fertility decline and subsequently decomposing these trends.

**Results:** We found that with the rising aggregate of the degree of fertility preference implementation index, continuous declining trends in demand for births and subsequent increases in the contribution made by either or both the wanted fertility and the degree of fertility preference implementation index across categories that fertility transition is certainly on course in all countries albeit at different levels, thanks to the family planning.

**Conclusions:** Family planning programs must therefore be accompanied by rigorous, consistent sensitization and public education.

## Introduction

Over the last decade, there has been considerable debate among scholars on fertility transition in Africa. Two conclusions emerge: slow pace of decline ( Bongaarts, 2017; Bongaarts & Casterline, 2013) because of weak facilitating social programs ( Otieno *et al*., 2016) and high demand for large families amidst weak family planning programs ( Bongaarts & Casterline, 2013). Accelerated fertility decline in Sub-Saharan Africa will occur if there is both substantial decline in desired fertility and increased level of preference implementation ( Bongaarts, 2017; Bongaarts & Casterline, 2013).

Despite these conclusions, there are also emergent exceptions, such as Rwanda, Ethiopia, Malawi ( Bongaarts, 2017; Mahy & Gupta, 2002). In a seminal workshop organized by Committee on Population of the National Academy of Sciences in 2015, there were suggestions that further search for explanations of fertility transition may lie in examinations of specific historical contexts of each population ( Casterline & Han, 2017). Our motivation for this study lies in this context. First, the East African countries share some common policy framework. Secondly, Rwanda and Kenya appear exceptional in the drive towards accelerating further fertility decline.

Kenyan fertility began to rapidly decline in the 1980s, followed by a stall (1998–2003) and then another phase of decline (2003–2014) recently ( KNBS, 2015). Tanzania progressed slowly in decline followed by stall mainly in rural areas ( Ezeh *et al*., 2009; Garenne, 2014) and then further decline within similar timelines as Kenya ( Otieno *et al*., 2016). Uganda has generally been regarded to still be at the pre-transition stage of fertility decline, recently exhibiting some modest declines ( Ezeh *et al*., 2009). Rwanda experienced rapid decline from 2005–2015 ( Dhillon & Phillips, 2015; May, 2017) because of the government’s management of the economy and provision of social and health services, including family planning, which are exceptional by regional standards ( Dhillon & Phillips, 2015; May, 2017). On the other hand Muhoza *et al.* (2014) revealed remarkable differences in desired and excess fertility between the four East African countries and between certain communities within these countries.

The main reason for focus on these countries is that any fertility change in one country may have implications in the neighboring country, because neighbouring regions share common dynamics, including language and cultural traits that permit shared flow of ideas to eventually spread new models of behavior. We analyze trends in two specific features that scholars have indicated will slow or increase in fertility decline. First, trends in fertility preferences among women and secondly, the extent to which women have been able to implement their fertility preferences during the course of fertility decline. Because of heterogeneity in fertility change within countries, we focus on trends by sub-national regions and social class stratification, since understanding the differences in fertility according to socioeconomic status and how these differences evolved over the fertility transition period is of great importance in understanding fertility decline. Conclusions about fertility change have been based on mostly cross-national comparisons based on national data but there has been less attention to sub national differentiation. It has been observed that enormous heterogeneity exists in Kenya, where the wealthy and higher educated people have fertility desires close to replacement level, regardless of religion, while poor, uneducated people, particularly those in Muslim communities, have virtually uncontrolled fertility ( Muhoza *et al*., 2014). On the other hand, in Rwanda the poor, uneducated people have the same desired fertility as their wealthy, educated compatriots, regardless of their religion.

## Conceptual framework

**Figure 1.**
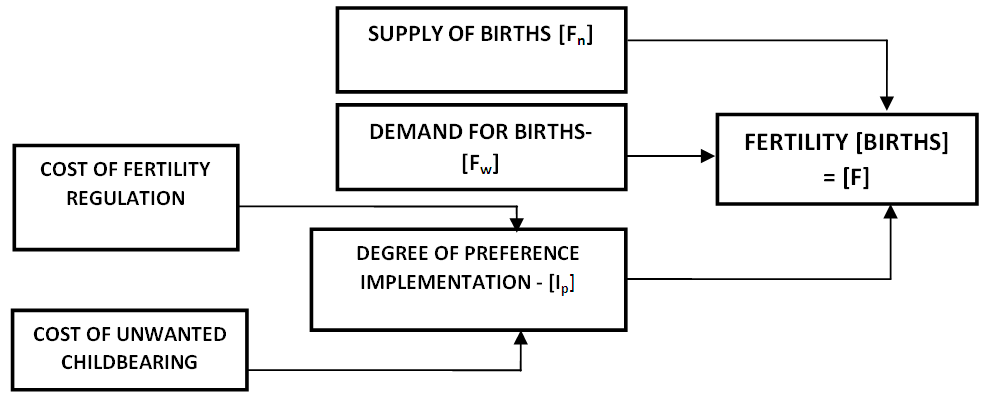
Key variables and interrelations in variant of supply-demand model. Source: Bongaarts (1993). The supply-demand framework for the determinants of fertility: An alternative implementation.

This study was guided by Bongaarts (1993) modification of the supply-demand framework for fertility analysis developed by Easterlin (1975) and Easterlin & Crimmins (1985). According to Bongaarts, Easterlin’s economic approach is a model of behavioral and biological factors affecting fertility in developing countries. The model consists of three fundamental concepts: demand for children, the potential supply of children, and the momentary and psychic costs of contraception. According to the model, women whose potential supply of births exceeds demand would consider contraception, taking into consideration the costs involved while choosing suitable family planning methods ( Montgomery, 1987). In this modification depicted in Figure 1, the fertility outcome measured by the total fertility rate is a function of: supply of births (natural fertility); demand for births (wanted fertility) and degree of preference implementation. The latter in turn is dependent on cost of fertility regulation and cost of unwanted childbearing. The degree of preference implementation is the net result of a decision-making process in which couples weigh the cost of fertility regulation and the cost of unwanted pregnancy.

## Methods

According to Bongaarts (1993), the relationship between these variables and fertility can be expressed as:

$$F_{o}=F_{w}+F_{u}\;(1)$$

Where F _u_ is unwanted fertility (which can simply be expressed as F _o_ – F _w_), and

$$\mathrm{Fu}=(F_{n}-F_{W}\text{)}\times (1-I_{p}\text{)}\;(2)$$

Where F _n_ is total natural fertility and I _p_ is the degree of fertility preference implementation index. I _p_ ranges from 0 to 1. With full preference implementation, I _p_ = 1 (which implies that F _u_ = 0 and F = F _w_) and I _p_ is 0 with no preference implementation (this implies a substantial level of unwanted childbearing and F = F _n_). Bongaarts (1993) indicated that F _n_ is not the same as in total fecundity used in the Bongaarts proximate determinants but is taken to mean fertility level achieved in absence of contraception (see Bongaarts, 1993). F _u_ is a function of the difference between supply and demand, and the degree of preference implementation. Substitution of Equation 2 from Equation 1 yields

$$F=F_{W}\times I_{p}+F_{n}\times \left(1-I_{p}\right)\;(3)$$

$$F_{n}=F/\text{C}\;(4)$$

Where C implies is an index ranging from 0 to 1 measuring the reduction in proportional of natural fertility attributable to deliberate birth control.

C=1−1.02×U(5)

Where U represents the proportion of married women who were practicing contraception at the time of survey. It is measured as the number of married women using contraceptive method to the total number of married women. Substitution of Equation 5 into Equation 4 gives an estimate of F _n_, while rearranging Equation 3 gives:

$$I_{p}=\left(F_{n}-F_{o}\right)/\left(F_{n}-F_{W}\right)\;(6)$$

## Decomposition of fertility trends

When estimates of observed, wanted and natural fertility, as well as the index of implementation are available for two successive points at time t _1_ and t _2_ in the same population ( Bongaarts, 1993), then a decomposition can be obtained to identify causes of fertility declines in specific populations ( Table 1). Again, following Bongaarts’ 1993 formulation, the following variables were used.

**Table 1.** Decomposition of fertility.

	Time 1 (t _1_)	Time 2 (t _2_)
Observed fertility	F _1_	F _2_
Natural fertility	F _n1_	F _n2_
Wanted fertility	F _w1_	F _w2_
Index of preference implementation	I _p1_	I _p2_

The decline in fertility between t _1_ and t _2_ is simply equal to F _1_ – F _2_, and this difference can be expressed as a function of the mediating variables by substitution of Equation 3.

$$F_{1}-F_{2}=\left[F_{w1}I_{p1}+F_{n1}\left(1-I_{p1}\right)\right]-\left[F_{w2}I_{p2}+F_{n2}\left(I_{p2}\right)\right]\;(7)$$

Since the emphasis here is on examining changes in fertility that result from changes in determinants, this equation can be rewritten as

$$\text{Δ}F=\text{Δ}{\text{F}}_{w}{\bar{\text{I}}}_{p}+\text{Δ}{\text{I}}_{p}\text{(}{\bar{\text{F}}}_{\text{w}}-{\bar{F}}_{\text{n}}\text{)}+\text{Δ}{\text{F}}_{n}\left(1-{\bar{I}}_{\text{p}}\right)\;(8)$$

Where ΔF, ΔF _w_, ΔF _n_ and ΔI _p_ represent absolute changes in F, F _w_, F _n_ and I _p_, respectively, and F _w_, F _n_, and I _p_ with bars represent the average values of F _w_, F _n_ and I _p_, respectively. Equation 8 conveniently divides the observed fertility decline ΔF into three components corresponding to each of the three determinants as indicated below ( Table 2)

**Table 2.** Contribution to fertility decline change.

Change in	Contribution to fertility change (ΔF)
Natural fertility ΔF _n_	$\text{Δ}{\text{F}}_{n}\text{(}1-{\bar{\text{F}}}_{\text{p}}\text{)}$
Wanted fertility ΔF _w_	$\text{Δ}{\text{F}}_{W}\times {\bar{I}}_{p}$
Index of implementation ΔI _p_	$\text{Δ}{\text{I}}_{p}\text{(}{\bar{F}}_{w}-{\bar{F}}_{n}\text{)}$

The above formulation shows that a change in wanted or natural fertility to the observed fertility decline depends on the average level of implementation index. Similarly, the fertility effect from a given change in the index of implementation depends on the average between natural and wanted fertility (F _n_ – F _w_) Table 1 and Table 2). The percentage contribution of each of the determinants to fertility decline can also be obtained by multiplying the ratio of change of each of the determinants to total fertility change by 100.

## Data

This study utilized cross sectional secondary data gathered overtime by the Demographic and Health Surveys (DHS) collected between 1988 and 2015 for Kenya, Rwanda, Uganda and Tanzania from women of reproductive age (15 to 49 years). DHS data is highly comparable across countries and has been shown to be of high quality. DHS data often computes first the total fertility rates based on data for the three years preceding the survey for age group 15–49 expressed per woman and second the total wanted fertility rates for the three years preceding the survey for age group 15–49 expressed per woman. Total wanted fertility rate is calculated in the same way as the total fertility rate, but only including wanted births. However, an alternative method is also to utilize the formula for the proportion of women age 40–49 in union who want no more births. According to Bongaarts, F _w_ can be obtained from the equation:

$$F_{\text{w}}=F^{\text{w}}+1.09-W_{\text{m}}\left(40-49\right)\;(9)$$

Where F ^w^ is the proportion of women who want more children and W _m_ (40–49) is the proportion of women in union aged from 40 to 49 who want no more births. Bongaarts, however, was cognizant of the fact that indeed there are limitations especially with the information on wanted fertility. The key limitations highlighted here are rationalization, involuntary and voluntary fertility limitation.

## Results

### Trends in Total fertility rate, wanted fertility rate and fertility preference implementation Index

**Figure 2.**
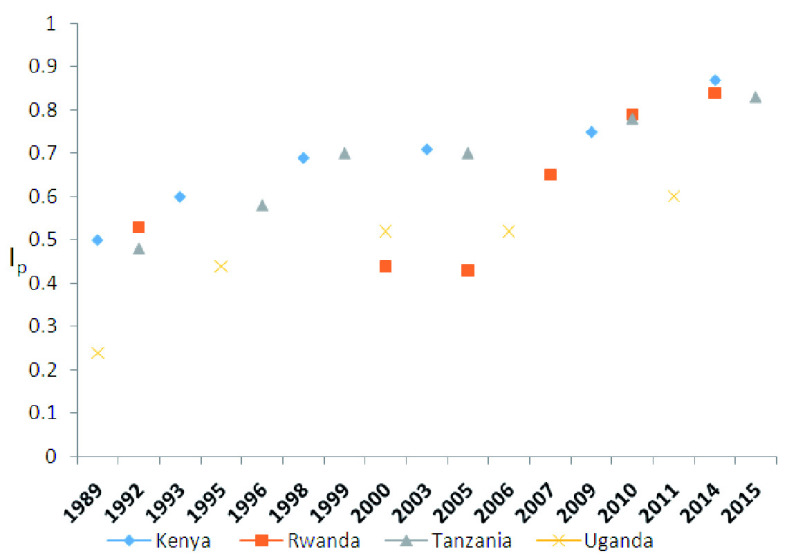
Trends in fertility implementation index (I _p_) since 1989.

Table 3, concerning the trends in total fertility rate, wanted fertility rate and fertility preference implementation index for East African countries, shows the results of the application of Bongaarts’ 1993 formula to estimate the degree of fertility preference implementation index for the four East African Countries: Kenya, Rwanda, United Republic of Tanzania and Uganda. Notably, all the countries experienced declines in both fertility and wanted fertility rates ( Figure 2). However, the highest change rate in decline occurred in Uganda. Subsequently, over the years as country fertility decline trends continue, Kenya and Rwanda have consistently registered the lowest wanted fertility rates, while Tanzania has since been surpassed by Uganda, as assessed by their respective last surveys. Wanted fertility rates for Tanzania have declined very slowly since 1996. Further, Rwanda recorded the highest rate of change in the desire to have fewer births compared to Kenya while Uganda also recorded the highest wanted fertility parameter values over the same periods until recently when it caught up with Tanzania in the course of decline.

**Table 3.** Trends in total fertility rate, wanted fertility rate and fertility preference implementation index for East African countries.

Country	Survey year	Total fertility rate	Total wanted fertility rate	Natural fertility rate	Preference implementation index
Kenya	2014	3.9	3.1	9.6	0.87
Kenya	2008–09	4.6	3.2	8.6	0.75
Kenya	2003	4.9	3.6	8.2	0.71
Kenya	1998	4.7	3.3	7.8	0.69
Kenya	1993	5.4	3.6	8.1	0.60
Kenya	1989	6.7	4.1	9.2	0.50
Rwanda	2014–15	4.2	3.3	9.2	0.84
Rwanda	2010	4.6	3.3	9.7	0.79
Rwanda	2007–08	5.5	3.8	8.8	0.65
Rwanda	2005	6.1	4.4	7.4	0.43
Rwanda	2000	5.8	4.6	6.7	0.44
Rwanda	1992	6.2	4.7	7.9	0.53
Tanzania	2015–16	5.2	4.5	8.6	0.83
Tanzania	2010	5.4	4.6	8.3	0.78
Tanzania	2004–05	5.7	4.8	7.8	0.70
Tanzania	1999	5.6	4.8	7.6	0.70
Tanzania	1996	5.8	4.8	7.1	0.58
Tanzania	1991–92	6.2	5.4	6.9	0.48
Uganda	2011	6.2	4.4	8.9	0.60
Uganda	2006	6.7	4.7	8.8	0.52
Uganda	2000–01	6.9	5.0	9.0	0.52
Uganda	1995	6.9	5.3	8.1	0.44
Uganda	1988–89	7.4	6.2	7.8	0.24

### Trends in the degree of fertility preference implementation

Figure 2 shows the general trends in the degree of fertility implementation index over the years for the respective countries. All countries have improved their implementation indices. Both Kenya and Tanzania experienced a plateau in I _p_ between 1998 and 2005 perhaps coinciding with the period of fertility decline. Contraceptive use also stagnated or stalled. Rwanda has experienced a rapid rise in I _p_ since 2005, which is consistent with the beginning of strength of family planning programs in the country. Uganda on the other hand is rising at a slower pace than its counterparts.

### Contribution of preference implementation and wanted fertility to fertility decline

This section presents the effect of both wanted fertility and preference implementation on fertility decline in the four countries based on the decomposition procedure. The analysis is restricted to the periods between 2000 and 2014. Positive values of wanted fertility, natural fertility and implementation index indicate contribution to decline while the negative values indicate tendency to increase fertility. Table 4, concerning the Estimated contribution of preference implementation and wanted fertility to fertility decline shows the change in fertility for the three countries over a period of about a decade. All the countries assessed have witnessed a decline in fertility since the beginning of the century. The highest decline in total fertility rate occurred in Rwanda, while the lowest decline occurred in Tanzania. All the countries subsequently witnessed a decline in wanted fertility rates, but the highest decline occurred in Rwanda. It is also in Rwanda where declines in the demand for children contributed to the highest decline in fertility. Similarly, ability to implement fertility desires has contributed to large declines in fertility across all the countries.

**Table 4.** Estimated contribution of preference implementation (I _p_) and wanted fertility (F _w_) to fertility decline.

Country	Survey year		Absolute decline in TFR	Absolute contribution to fertility decline			Percent contribution to fertility decline		
				F _w_	F _n_	I _p_	F _w_	F _n_	I _p_
Kenya	2003	2014	1	0.451	-0.289	0.838	45.1	-28.9	83.8
Rwanda	2005	2014	1.89	0.692	-0.642	1.843	36.6	-33.9	97.4
Tanzania	2004	2015	0.49	0.207	-0.176	0.459	42.2	-36	93.8
Uganda	2000	2011	0.68	0.314	0.026	0.342	46	3.9	50.1

TFR, total fertility rate; Fn, total natural fertility.

Except for Uganda, in all the other countries, the ability to implement fertility preference contributed to nearly twofold decline in fertility. The results complement the assertion by Bongaarts & Casterline (2013) and Casterline & Han (2017) that acceleration of fertility decline occurs when both demand for children and ability to implement fertility desires occur simultaneously, as in the case of Rwanda and to some extent Kenya. However, the national data mask within country differences. An example is why Tanzania never experienced any large declines in the contribution of wanted fertility to the course of fertility decline as with the case of Kenya and Rwanda. 

### Sub-national variations in contribution of preference implementation and wanted fertility to fertility decline

Table 5, on the estimated I _p_ and contribution of wanted fertility and preference implementation to fertility change by region, presents variations in fertility preference implementation index and the contribution of wanted fertility and preference implementation to fertility decline at sub-national levels for the four countries. In Kenya, most regions have I _p_ above 0.85 except for North Eastern region with negative low value. Regions in Rwanda have I _p_ ranging 0.75 in Western region to 0.96 in Northern region. Tanzania has very wide variation in I _p_, with the lowest (0.44) in Pemba south to the highest (1.00) in Kilimanjaro region. However, some regions experience unique results (Dar es Salam, Pwani, Lindi, Mtwara and Zanzibar Town west). Such results might arise due to measurement errors in wanted fertility rate or issues in the measurement of contraceptive use that is utilized in the estimation of natural fertility. However, the low fertility of Mtwara region did not arise from high contraceptive use but from long post-partum amenorrhea. Thus, it may be the case of the effects of other proximate determinants may also influence the estimation of I _p_. 

**Table 5.** Estimated preference implementation index (I _p_) and contribution of wanted fertility (F _w_) and preference implementation to fertility change by region.

Country	Region	Estimated I _p_ for latest survey	Absolute change in				Percent contribution to change in TFR		
			TFR	F _w_	F _n_	I _p_	F _w_	F _n_	I _p_
**Kenya**	Nairobi	1.00	0.0	0.2	-1.7	0.21	24	-206	-56
	Central	1.00	0.6	-0	-0.5	-0.13	-10	-51	70
	Coast	0.96	0.6	0.3	-1	-0.22	26	-83	48
	Eastern	0.95	1.4	0.4	-1.9	-0.25	33	-155	139
	Nyanza	0.85	1.3	0.7	-2.4	-0.35	48	-165	160
	Rift Valley	0.85	1.3	0.3	-0.9	-0.32	21	-61	136
	Western	0.87	1.1	0.3	-3.2	-0.31	21	-225	180
	North Eastern	-0.09 *	0.6	1.8	0.4	0.08	-8	-2	28
**Rwanda**	Kigali	0.90	0.7	1.1	-0.2	-0.65	64	-11	225
	South	0.83	1.6	1	-0.8	-0.62	52	-39	234
	West	0.75	2.0	2.2	0.4	-0.09	155	29	33
	North	0.96	2.7	1.7	-0.6	-0.31	137	-51	115
	East	0.78	1.9	2.8	-0.4	-0.09	204	-26	35
**Tanzania**	Tabora	0.66	0.6	-2.0	-1.8	0.15	-132	-133	-40
	Shinyanga	0.65	1.2	0.0	1.8	0.04	-13	119	-14
	Kigoma	0.68	0.5	-1.0	0.7	0.22	-87	54	-67
	Kilimanjaro	1.00	0.5	1.2	0.8	-0.54	100	62	148
	Tanga	0.90	0.3	0.9	3.2	-0.27	69	249	87
	Dodoma	0.88	1.1	-1.0	-4	0.25	-121	-399	-72
	Singida	0.75	-0.4	0.2	-2.5	0.44	20	-248	-173
	Mbeya	0.95	1.7	0.2	1.1	-0.03	19	101	14
	Iringa	0.81	0.3	1.0	1.9	0.44	103	196	-137
	Rukwa	0.69	0.5	-1.0	-2.3	0.54	-67	-217	-204
	Kagera	0.90	1.8	-1.0	0	0.02	-100	4	-10
	Mwanza	0.56	0.3	0.0	-0.6	0.40	-30	-44	-96
	Mara	0.64	0.3	0.0	-1.2	0.08	0	-81	-32
	Dar es Salaam	1.13 **	-0.8	0.1	2.4	-0.24	10	241	63
	Pwani	1.20 **	0.6	0.6	1.4	-0.73	50	117	216
	Morogoro	0.92	0.0	1.1	-1.2	-0.50	73	-81	187
	Lindi	1.25 **	0.3	0.9	0.0	-0.67	83	2	207
	Mtwara	1.23 **	1.3	2.1	0.3	-0.32	226	32	67
	Ruvuma	0.92	0.6	1.8	-1.0	0.12	177	-103	-45
	Arusha	0.96	0.2	0.5	-2.6	-1.76	4	-19	135
	Manyara	0.72	0.4	2.8	-0.2	3.61	708	-58	-623
	Zanzibar North	0.72	-0.1	0.0	-2.03	-0.5	0.0	-289	100
	Zanzibar South	0.93	-1.3	0.1	-4.45	-0.1	34	-208	66
	Town West	1.09 **	0.5	0.2	-0.54	-0.25	137	-13	-37
	Pemba North	0.86	0.0	-0.1	-0.55	-0.15	135	198	-35
	Pemba South	0.44	0.6	-0.1	0.56	-0.15	69	-671	31
**Uganda**	Central	0.84	0.6	0.0	0.7	-0.17	-15	51	59
	Eastern	0.52	0.1	0.0	-1.4	-0.25	-8	-57	105
	Western	0.59	0.6	0.4	-0.6	-0.21	19	-27	70
	Northern	0.51	1.4	0.4	1.5	-0.12	18	68	39

Base year: Kenya, 2003; Rwanda, 2005; Tanzania, 2004; Uganda, 2000. Latest survey: Kenya, 2014; Rwanda, 2014-15; Tanzania, 2014-15; Uganda, 2011. *I _p_ < 0; **I _p _> 1. TFR, total fertility rate; F _n_, total natural fertility.

The largest declines in fertility within the decade occurred in Regions of Rwanda (North (2.7-fold), West (2-fold) and East (1.9-fold)). This was followed by Kagera (1.8-fold) and Mbeya (1.7-fold) regions of Tanzania. The next highest decline occurred in South region of Rwanda, Northern of Uganda and Eastern regions Kenya followed by Mtwara region of Tanzania, Nyanza and Rift valley regions of Kenya. Most regions of Tanzania had minimal or no decline at all over the period assessed. The Nairobi region of Kenya, Morogoro and Pemba North regions of Tanzania equally registered no significant change in fertility ( Table 3). What is notable is that some regions in Tanzania actually registered increases in fertility. These include the largest urban centre Dar-es-Salaam (−0.8-fold) and Singida (−0.4-fold) in Tanzania mainland and Zanzibar North (−0.7-fold), and Zanzibar South (−1.3-fold) in Zanzibar Island. The low decline in fertility rate as well as observed increase in fertility in some regions of Tanzania may explain why there has been slow change in average fertility indicators for Tanzania at the national level. Fertility preference is by large a key contributor to the reduction of fertility, notably in Tanzania. The regions with largest contribution by change in wanted fertility to fertility decline were Manyara at 708% and Mtwara at 226%. This was followed by the East region of Rwanda at 204%, Ruvuma of Tanzania at 177% and the West and North regions of Rwanda with 155% and 137%, respectively. Others with significant contributions were Iringa, Kilimanjaro and Lindi regions of Tanzania. Thus, the major contribution to fertility decline in Tanzania across many sub-regions is the change in fertility preferences.

In Kenya, Rwanda and Uganda, the ability to implement fertility desires I _p_ was the greatest contribution to fertility decline for most of the regions. The contribution of I _p_ was highest in South and Kigali regions in Rwanda, Pwani and Lindi regions of Tanzania, where I _p_ contributed to over a twofold decline in fertility. Notwithstanding this, the I _p_ contributed least to fertility decline mostly in regions of Tanzania. These regions include Manyara, Rukwa and Iringa. Bongaarts’ formulation equation rule is that the index of implementation of fertility can only fall between zero and one. However, this has not been the case in some segments of the populations within this study. It calls for a reflection on some regions that registered erratic indices. These regions actually posted indices values either below zero or above unity. The regions were Pemba South of Tanzania North Eastern region of Kenya at −0.09-fold, while the indices beyond unity values were the Pwani, Mtwara, Lindi and Dar es Salaam in Tanzania, which therefore calls for revisiting the formula to include the effect of other related proximate determinants of fertility decline, acting as a normalization of sort.

### Socio economic differences in contribution of wanted fertility and preference implementation to fertility decline

Table 6 highlights the contributions made by the wanted fertility and the degree of fertility preference implementation index to the change in fertility based on two key socioeconomic variables namely, the type of place of residence and the level of educational attainment of the women. In all regions, I _p_ increased with increase in level of education. I _p_ was also higher in urban areas compared to rural areas in all regions. In Kenya and Rwanda, fertility decline was higher in rural areas and among women with lower or no education. Tanzania posted mixed and unique results. In general, the degree of fertility preference implementation index was higher among women of urban resident as well as among women with higher education. 

**Table 6.** Contribution of preference implementation index (I _p_) to fertility decline by place of residence and education.

Country	Characteristic	Latest I _p_	Change in TFR	Contribution to change in TFR			% Contribution to change in TFR		
				F _w_	F _n_	I _p_	F _w_	F _n_	I _p_
**Kenya**	Urban	0.90	0.2	0.0	-1.8	-0.13	0	-151	51
	Rural	0.82	0.9	0.5	-1.7	-0.21	35	-118	103
	None	0.75	0.2	0.0	-0.4	-0.38	-22	-23	60
	Primary	0.83	1.1	0.6	-2.4	-0.26	42	-168	140
	Secondary	0.89	0.3	0.0	-1.3	-0.12	-8	-105	60
	Higher	0.91	0.0	0.0	1.0	-0.00	-9	93	0
**Rwanda**	Urban	0.81	1.3	0.9	-1.3	-0.3	60	-84	113
	Rural	0.78	2.0	1.6	-1.3	-0.49	85	-70	175
	None	0.74	1.8	1.5	-1.4	-0.52	71	-66	181
	Primary	0.79	1.6	1.3	-2.2	-0.48	71	-120	188
	Secondary	0.84	1.3	0.9	0.0	-0.20	67	-4.	65
	Higher	0.90	0.1	0.0	0.1	-0.08	-17	6.0	26
**Tanzania**	Urban	0.84	-0.2	0.0	-0.4	-0.05	-25	-32	12
	Rural	0.75	0.5	0.5	-1.0	-0.18	33	-66	51
	None	0.76	0.0	0.0	-1.6	-0.29	0	-101	62
	Primary	0.78	0.2	0.2	-0.9	-0.09	15	-66	29
	Secondary	0.87	-0.1	0.0	-0.2	-0.05	-17	-19	10
	Higher	0.85	-0.4	0.0	1.2	0.09	-36	104	-18
**Uganda**	Urban	0.81	0.6	0.4	0.7	-0.04	31	53	14
	Rural	0.56	0.3	0.4	-0.5	-0.11	20	-27	37
	None	0.45	0.8	0.9	0.3	-0.1	36	12	27
	Primary	0.57	0.4	0.3	-0.3	-0.11	15	-17	41
	Secondary	0.77	-0.5	-1.0	-1.1	-0.04	-37	-79	14
	Higher	0.78	0.2	0.4	-0.3	0.01	32	-27	-4

Base year: Kenya, 2003; Rwanda, 2005; Tanzania, 2004; Uganda, 2000. Latest survey: Kenya, 2014; Rwanda, 2014-15; Tanzania, 2014-15; Uganda, 2011. TFR, total fertility rate; Fn, total natural fertility; F _w_, wanted fertility.

In Rwanda, both changes in wanted fertility and ability to implement fertility desires contributed to fertility decline across all the sub groups. However, in Kenya it was the ability to implement fertility desires that made major contribution to fertility decline across most of the subgroups. Uganda had almost similar results to Rwanda except that the contribution was lower. Similarly, Tanzania had similar results to Kenya, though with lower contribution values.

## Discussion

In this study, we focused on the sub-national regions (provinces) of East African countries, analyzing trends in fertility preferences among women and the extent to which these women have been able to implement these fertility preferences during the course of declining fertility in these countries. The main reason was that fertility change in one country may have implications in the neighboring country because neighboring regions share common language and cultural traits that permit shared flow of ideas and eventual spread of new models of behavior. In Kenya and Rwanda, the most important contributor to fertility decline by region, place of residence and socio-economic groups is the ability to implement fertility desires (I _p_). The same is true for Uganda; however, Tanzania posted mixed results by different groups.

Rwanda has had the largest fertility decline due to the contributions by both changes in the wanted fertility and ability to implement fertility desires. The results highlight one core result: fertility declines faster when both wanted fertility and ability to implement desired fertility occur simultaneously, as in the case of Rwanda. Secondly, we find enormous heterogeneity in fertility change within countries. The largest heterogeneity existed in Tanzania and lowest in Rwanda. As indicated by Muhoza *et al*. (2014), there are not only remarkable differences in desired fertility between the four East African countries and between certain communities within these countries, but also the degree to which the different communities are able to implement their desires. These differences may reflect cultural differences in reproductive behavior, as well as effect of development and population density which create resource needs pressures. For example, for Kenya, Tanzania and Uganda, regions with high agricultural potential, high population density and the highest densities of development inputs, have high implementation indexes and the lowest desired family sizes. 

The results here concur with Bongaarts & Casterline (2013), who found that although each country has a unique fertility trajectory, fertility transitions share similarities. Countries typically have high and relatively stable fertility during the pre-transitional period which comprises most of human history ( Bongaarts & Casterline, 2013). Once the transition starts, the pace of fertility decline in the first one or two decades following the onset is usually faster than in later decades. In any given country, today’s level of fertility may be a function of the pre-transitional level of fertility, the timing of the onset of the fertility transition, and the pace of fertility change. As noted earlier, conventional transition theory predicts that fertility levels are inversely related to socioeconomic development indicators ( Bongaarts & Casterline, 2013).

There is some caution that may be made in the estimation of I _p_ as evident in the North Eastern region of Kenya and some regions of Tanzania. The I _p_ is dependent on development of a region, but more importantly closely associated with extent of unwanted fertility and hence unmet need for contraception. A close assessment into the cultural factors reveal that Muslim dominated areas may appear to have low degree of I _p_. However, this may be confounded by other factors. Zanzibar Town West region has a high I _p_ while Pemba south has low I _p_, whereas the North Eastern region of Kenya has low levels of contraception utilization. These regions—dominated by cultural dogmas and doctrines, especially religious beliefs—exhibit rationalized feedbacks and indeed prone to even non-numeric responses ( Casterline & Han, 2017). Furthermore, the level of I _p_ may be dependent on program reach, confirming that fertility change must be based on the concurrent change in both women’s preferences and ability to implement those preferences.

## Conclusions and recommendations

It is now clear that with the rising aggregate level of the degree of fertility preference implementation index, continuous declining trends in demand for births and the subsequent increases in the contribution made either singly or jointly by the wanted fertility and the degree of fertility preference implementation index across the various categories indicates that fertility transition is certainly on course in all countries albeit at different levels. In sum, the evidence examined here shows that the pattern of fertility decline in the region is indeed unique. In addition, the relatively slow pace and level of development in the region implies that the cost of raising children has remained low compared to other developing regions and the benefits of having offspring remain substantial in the subsistence economies, which characterizes the East African countries. This is also true of a majority of the Sub-Saharan African countries in general ( Bongaarts, 2017).

The dominant mediums of diffusion of knowledge and information among populations, such as the public media and urbanization, though still very low, are expected to increase their influence. The future pace of fertility decline in the region will therefore likely be slower than the pace of decline in other regions at comparable times from the transition onset, unless special interventions are undertaken ( Bongaarts & Casterline, 2013). This is a result of the duration it has taken the region to get to where it is compared to the countries that went through transition in the earlier years.

There is, however, an additional policy option to accelerate fertility decline. Investing in family planning programs to provide information about and access to contraception in order to permit control of reproductive lives and avoiding unplanned pregnancies. These countries in general are characterized among the ones with low contraception ( Alkema *et al*., 2013). The key cause of an unmet need for contraception is that contraception is either unavailable or often too costly to the consumers and sometimes the population is ignorant of the existence or usage of contraception. In addition, there are significant non-economic costs such as health concerns, social disapproval, and spousal resistance, as well as unnecessary medical barriers requiring higher-level expertise for utilization ( Casterline & Sinding, 2000). The unmet need is responsible for most of the unsatisfied demand subsequently aggravating unplanned pregnancies.

These family planning programs therefore must be accompanied by rigorous, consistent sensitization and public education campaigns through media among other modes of communication, which in turn trigger demand for contraception in anticipation of lower desired family size by diffusing new ideas about the benefits of smaller families and the role of women ( Bongaarts, 2017). The degree of implementation is expected to take in values between zero and one. However, there is evidence of values greater than one as well as negative values. This points to a limitation in the frontiers of knowledge with regards to this study. Due to these glaring violations further research is recommended to assess how well the fertility preference implementation index is suited to measuring fertility success.

## Data availability

### Source data

The datasets analyzed during the current study are available in the MEASURE DHS repository ( http://www.measuredhs.com). Access to the dataset requires registration and is granted to those that wish to use the data for legitimate research purposes. A guide for how to apply for dataset access is available at: https://dhsprogram.com/data/Access-Instructions.cfm. The DHS datasets used in the present study are listed in Table 3.
