# Supplementary material for: Trends in fertility preference implementation among selected Eastern African countries
Source: F1000Res. 2020 Feb 3;9:79. [Version 1] doi: 10.12688/f1000research.22064.1 (PMC9020534; doi:10.12688/f1000research.22064.1)
Supplement: Peer review history

# Peer review: Reviewer response for version 1

**DOI:** 10.5256/f1000research.24334.r128259

**Published:** 2022-04-19

**Authors:** Collins C. Iwuji

**Affiliations:** 1 University of Sussex, Brighton, UK; 2 Africa Health Research Institute, KwaZulu-Natal, South Africa

This study investigated the interaction between wanted fertility and fertility preference implementation in shaping fertility trends in 4 countries in Eastern Africa. This is a detailed analysis that explored this phenomenon both at the national and subnational level highlighting heterogeneity within and between countries.

**Abstract**

- The sentence summarising the results in the abstract is very long and difficult to follow. I suggest the authors revise this and use shorter sentences for clarity

**Main article**

**General comments**

- The authors should be consistent when describing the different types of fertility – wanted fertility, natural fertility, and total fertility – and should use consistent notations throughout the article. This makes for ease of reading and understanding.

**Results**

- The first paragraph in the results section where Figure 2 was mentioned should have been Table 3.
- The authors did well to explore other socio-economic factors that could explain the observed fertility trend in the four countries studied, such as place of residence and educational attainment.
- In the analysis or perhaps in the discussion, other country-level factors that could shape decisions around fertility should have been explored, for example, whether education is free in the countries and the availability of free health care. I suggest the authors explore these in the discussion.

Is the work clearly and accurately presented and does it cite the current literature?

Yes

If applicable, is the statistical analysis and its interpretation appropriate?

Yes

Are all the source data underlying the results available to ensure full reproducibility?

Yes

Is the study design appropriate and is the work technically sound?

Yes

Are the conclusions drawn adequately supported by the results?

Yes

Are sufficient details of methods and analysis provided to allow replication by others?

Yes

Reviewer Expertise:

Clinical epidemiology, population health, HIV, STIs, planetary health

I confirm that I have read this submission and believe that I have an appropriate level of expertise to confirm that it is of an acceptable scientific standard.

# Peer review: Reviewer response for version 1

**DOI:** 10.5256/f1000research.24334.r63692

**Published:** 2020-06-10

**Authors:** John N. Bongaarts

**Affiliations:** 1 Population Council, New York, NY, USA

The paper sheds light on the causes of fertility transition in selected Eastern African countries. The authors rely on a decomposition method I proposed in 1993 which estimates the roles of three factors in fertility decline: 1) decline in wanted fertility, 2) increase in preference implementation and 3) change in natural fertility. Results show that the rise in the implementation index is the main driver of fertility decline. Although the study does not directly assess the impact of family planning programs, the authors note that these programs aim to help women in implementing their reproductive preferences and disseminate information about the benefits of contraception and smaller families.

 The text is clearly written and the analysis is technically competent. Two minor edits:

1. The total fertility rate is represented by three different abbreviations: TFR, F, and Fo. This is confusing and should be fixed.
2.  The first paragraph of the results section makes reference to Figure 2. This should be Table 2.

Is the work clearly and accurately presented and does it cite the current literature?

Yes

If applicable, is the statistical analysis and its interpretation appropriate?

Yes

Are all the source data underlying the results available to ensure full reproducibility?

Yes

Is the study design appropriate and is the work technically sound?

Yes

Are the conclusions drawn adequately supported by the results?

Yes

Are sufficient details of methods and analysis provided to allow replication by others?

Yes

Reviewer Expertise:

Demography, Fertility, Family planning

I confirm that I have read this submission and believe that I have an appropriate level of expertise to confirm that it is of an acceptable scientific standard.
